# Perceived Severity of Cyberbullying: Differences and Similarities across Four Countries

**DOI:** 10.3389/fpsyg.2017.01524

**Published:** 2017-09-20

**Authors:** Benedetta E. Palladino, Ersilia Menesini, Annalaura Nocentini, Piret Luik, Karin Naruskov, Zehra Ucanok, Aysun Dogan, Anja Schultze-Krumbholz, Markus Hess, Herbert Scheithauer

**Affiliations:** ^1^Department of Educational Sciences and Psychology, University of Florence Florence, Italy; ^2^Institute of Computer Science, University of Tartu Tartu, Estonia; ^3^Department of General Education, Institute of Education, University of Tartu Tartu, Estonia; ^4^Psychology Department, Hacettepe University Ankara, Turkey; ^5^Psychology Department, Ege University Izmir Izmir, Turkey; ^6^Department of Education and Psychology, Freie Universität Berlin Berlin, Germany

**Keywords:** cyberbullying, perceived severity, adolescents, ESEM, exploratory structural equation modeling, cross-national comparison

## Abstract

Cyberbullying is a ubiquitous topic when considering young people and internet and communication technologies (ICTs). For interventional purposes, it is essential to take into account the perspective of adolescents. This is the reason why our main focus is (1) investigating the role of different criteria in the perceived severity of cyberbullying incidents, and (2) examining the differences between countries in the perceived severity of cyberbullying. The sample consisted of 1,964 adolescents (48.2% girls) from middle and high schools of four different countries, i.e., Estonia, Italy, Germany, and Turkey. The participants' age ranged from 12 to 20 years old with a mean age of 14.49 (*SD* = 1.66) years. To assess perceived severity, participants rated a set of 128 scenarios, which systematically included one or more of five criteria (intentionality, repetition, imbalance of power, public vs. private, and anonymity) and represented four types of cyberbullying behaviors (Written—Verbal, Visual, Exclusion, Impersonation). The role of different criteria was analyzed using the Exploratory Structural Equation Modeling (ESEM). Results showed a similar structure across the four countries (invariant except for the latent factors' means). Further, criteria of imbalance of power and, to a lesser extent, intentionality, anonymity, and repetition always in combination, were found to be the most important criteria to define the severity of cyberbullying. Differences between countries highlighted specific features of Turkish students, who perceived all scenarios as more severe than adolescents from other countries and were more sensitive to imbalance of power. German and Italian students showed an opposite perception of anonymity combined with intentionality. For Italian participants, an anonymous attack was less threatening than for participants of other countries, whereas for German students anonymity caused more insecurity and fear. In addition, Italian adolescents were more perceptive of the criterion of intentionality. Finally, Estonian adolescents did not show strong differences in their factor scores compared to adolescents from the other countries.

## Introduction

Cyberbullying has been designated as a serious public health problem, which can dramatically impact the lives of adolescents. On average about 15% of adolescents may be victimized online (Modecki et al., 2014). How do adolescents perceive this problem? Are some attacks more or less serious for them? The aim of this study is to address adolescents' perceived severity of cyberbullying in different countries based on different criteria applied to hypothetical scenarios.

Cyberbullying has been defined as a modern form of bullying accomplished through electronic forms of contact (e.g., SMS, Facebook, YouTube; Smith et al., 2008; Tokunaga, 2010; Menesini et al., 2013). In this definition, scholars have stressed the role of *intentionality, repetition*, and *imbalance of power* as three main criteria shared with traditional bullying (Slonje and Smith, 2008; Vandebosch and Van Cleemput, 2008; Dooley et al., 2009; Slonje et al., 2013). Intentionality refers to the perpetrator's motives and is supposed to differentiate between deliberate harm and unintended hurtful action. Repetition differentiates (cyber)bullying from single aggressive acts and helps to classify this type of behavior into a specific sub-category of aggression (Dooley et al., 2009). The imbalance of power refers to power abuse between the perpetrator and the target, leaving victims helpless and unable to defend themselves.

Menesini et al. (2012) investigated the effect of these three dimensions of this definition of cyberbullying among adolescents from six countries and found that imbalance of power was the most important factor, followed by intention to do harm and then repetition. Specifically, the latter seems to be the least relevant characteristic and it may have a different impact with regard to direct and indirect forms of cyberbullying (Langos, 2012). Some studies also added two other specific features, such as *anonymity* and *publicity*, related to the online environment (Nocentini et al., 2010). Anonymity because the victims often do not know the identities of their bully(ies) (Slonje and Smith, 2008; Dooley et al., 2009). Publicity because the online attacks usually occurs in a public context (Cuadrado-Gordillo and Fernández-Antelo, 2016). Considering anonymity and publicity, previous studies (Nocentini et al., 2010; Menesini et al., 2012) suggested that these criteria are not a prerequisite for labeling an action as cyberbullying, though they are relevant to determine the severity of the attack.

Besides these criteria, some studies have analyzed the types of attacks and how adolescents themselves might perceive them. Several authors refer to Willard's (2007) classification of different online behaviors, such as flaming, harassment, denigration, impersonation, outing, trickery, exclusion, and cyberstalking. Nocentini et al. (2010) proposed and experimentally confirmed (Palladino et al., 2015) a more parsimonious model consisting of four typologies: (1) written-verbal behaviors (i.e., phone calls, text messages, chats, social networks etc.); (2) visual behaviors (i.e., posting, sending or sharing compromising pictures, and videos); (3) exclusion (i.e., excluding someone from an online group on purpose); and (4) impersonation (i.e., using another person's name and account to damage him or her).

Although, extensive research has been carried out on different aspects of cyberbullying definition and involvement, there is still little agreement on the general definition of this problem, with relevant implications on its measurement and evaluation. This consideration derives from literature on traditional bullying where several authors request a clearer definition of bullying and cyberbullying. From a theoretical point of view, some accounts describe bullies as individuals who lack social skills, have a low self-esteem, deficiencies in social information processing, and other adjustment problems. Others see bullying as a functional and adaptive behavior associated with benefits and rewards related to the dominant position (Menesini and Salmivalli, 2017). These two different approaches imply different definitions as reported by Volk et al. (2017).

The present study is focused on perceived severity which might contribute to the definition but it does not coincide with it. According to Chen et al. (2015), the perceived severity refers to an individual's implicit perception of the potential and practical harm of the behavior to themselves or to others.

Individuals may perceive the severity of cyberbullying behavior based on its negative impact, type of behavior and defining criteria such as level of intentionality, imbalance of power and other dimensions related to the attack.

Among the very few studies on perceived severity of cyberbullying, an important contribution has been given by Sticca and Perren (2013), who investigated the relative importance of the medium (traditional vs. cyber), and of criteria of publicity (public vs. private), and anonymity (anonymous vs. not anonymous). They found out that public scenarios were perceived as worse than private ones. Additionally, they also discovered that anonymous scenarios were perceived as worse than those perpetrated by known persons. Chen and Cheng (2016), in another study involving 707 students in Taiwan, confirmed that cyberbullying behaviors happening in private context were rated as less severe than those occurred in public context.

The role of anonymity was also addressed by Bryce and Fraser (2013), who pointed out other relevant processes, i.e., the mechanisms of disinhibition and inability to view the direct impact of victimization. Other studies did not find differences in relation to anonymity and publicity, but to the nature of the incident (Bauman and Newman, 2012; Chen et al., 2015). Specifically, physical and verbal bullying was perceived as more severe than relational bullying and cyberbullying. The results of another study supported previous findings on the role of imbalance of power, anonymity, and publicity in moderating the perceived severity of cyberbullying (Dredge et al., 2014).

Although, specific literature on the perceived severity of cyberbullying is scarce, several studies have highlighted the severity of this behavior focusing on its negative consequences (Ortega et al., 2012). A recent meta-analysis (Kowalski et al., 2014), showed that individuals who engaged in cyberbullying reported use of drugs and alcohol, and problems of conduct and school achievement. In the case of the victims, several positive relationships between cybervictimization and psychological symptoms such as anxiety, depression, loneliness, emotional problems, somatic symptoms, and suicidal ideation were found (Bauman et al., 2013; Gini and Espelage, 2014).

Evidence presented in the literature on incidence and long-term consequences of cyberbullying suggests that this is a very serious problem and can have detrimental effects both on the victims and the bullies. However, literature on perceived severity is limited by the fact that most studies have not explicitly and systematically addressed this aspect. Rather, they merely addressed one single criterion at a time and not the combination or the interaction between different criteria, which is to be expected given the complexity of cyberbullying.

If we consider differences between countries, the literature on the definition of cyberbullying does not show significant effects related to the country of origin. For instance, a cross-national study (Menesini et al., 2012) and single country studies (Naruskov et al., 2012; Schultze-Krumbholz et al., 2014) showed general support for the structural equivalence of the dimensions used by adolescents across six different European countries (i.e., Italy, Germany, Spain, France, Estonia, and Sweden). Conversely, a focus group study (Nocentini et al., 2010), involving Italy, Germany and Spain revealed some differences. For instance, to define cyberbullying, German and Spanish participants considered intentionality as a relevant factor, whereas Italian girls stressed the importance of the victim's feelings. In all three countries, anonymity was important due to the impact it might have on the victim. Looking at previous literature, studies on the perception of severity are scarce and restricted to western countries where the level of ICTs used has been present for several years. In Turkey, on the contrary, the use of new technologies by children and adults is still at an initial stage. According to d'Haenens and Ogan (2013), Turkish adolescents are the most digitally unequal among adolescents from several EU countries (e.g., Austria, Germany, the Netherlands), with regard to computer ownership and computer skills. Although, research on cyberbullying in Turkey has recently increased and has become a major concern among school professionals, teachers, and parents, relatively little is known about children in terms of cross-cultural comparison. Findings related to traditional forms of bullying have consistently shown that bullying/victimization is a common and frequent experience, while the prevalence of cyberbullying is lower with a frequent overlap between the two forms (Bayar and Ucanok, 2012).

In summary, given the differential impact of ICT and cyberbullying in different countries, it is relevant to compare historical EU countries, such as Italy and Germany, with a country that recently joined the EU (i.e., Estonia) and with another country that wishes to enter the EU (i.e., Turkey).

### The present study

The present study investigates adolescents' perception of cyberbullying focusing on the construct of perceived severity across different countries.

Specifically, there are two primary aims:

1. Investigating the role of different criteria (intentionality, repetition, imbalance of power, anonymity, and publicity) for the perceived severity of cyberbullying incidents, and
2. Examining differences between countries.

## Materials and methods

### Participants

The study was part of a cross-national study originally developed within the European project COST Action IS0801 “Cyberbullying: coping with negative and enhancing positive uses of new technologies, in relationships in educational settings.” The participants were 1,964 adolescents (48.2% girls) from middle and high schools of four different countries: Italy, Germany, Estonia, and Turkey (see Table 1 for descriptive data). The age range was 12–20 years with a mean age of 14.49 (*SD* = 1.66) years. Between 327 (Italy) to 631 (Turkey) students participated in the study in each country.

**Table 1:** Sample characteristics.

	**N (%)**	**Gender**		**Grade**	**Age Mean (SD)**
		**Male (%)**	**Female (%)**		
Estonia	440 (22.4%)	216 (49.1%)	224 (50.9%)	6 (*N* = 227); 9 (*N* = 213)	14.72 (1.57)
Germany	566 (28.8%)	302 (53.4%)	264 (46.6%)	7 (*N* = 5); 8 (258) 9 (*N* = 13); 10 (290)	14.68 (1.35)
Italy	327 (16.6%)	187 (57.2%)	140 (42.8%)	7 (*N* = 138); 10 (*N* = 189)	15.13(1.90)
Turkey	631 (32.1%)	313 (49.6%)	318 (50.4%)	6 (*N* = 198); 7 (*N* = 159) 9 (*N* = 146); 10 (*N* = 128)	13.84 (1.62)
Total	1964	1018 (51.8%)	946 (48.2%)	6 (N = 425) 7 (*N* = 302) 8 (*N* = 258) 9 (*N* = 372) 10 (*N* = 607)	14.49 (1.66)

### Procedure

The assessment in each country took place during the school year 2013–2014. The authors were responsible for data collection. Trained researchers administered questionnaires to the students during school time. In Germany, the questionnaires were administered online during regular course time; in the other countries, the questionnaires were filled in using paper and pencil.

In each country, in agreement with the national law, the consent procedure for the study consisted of an approval by the schools and of an active parental consent when requested. Specifically, in Italy and Estonia, the active consent of the school, parents and students was obtained prior to questionnaire administration. In Germany, active parental consent was acquired for students under 14 (in accordance with the Senate regulations for empirical studies in the school setting) and students' active consent was collected in the first step of the questionnaire. In Turkey, the research team requested a specific permission from the Ministry of Education and from school administrations. Participation was entirely voluntary and anonymous. Participants were informed that they could withdraw from the study at any time.

### Instruments

We developed a revised version of the Scenarios Questionnaire (Menesini et al., 2012) to assess the perceived severity of different cyberbullying scenarios. This complex and comprehensive questionnaire provides a set of 32 scenarios, which systematically combined the presence or absence of five criteria—namely Anonymity, Imbalance of power, Repetition, Public vs. Private and Intentionality—in all possible combinations (from none to every criterion being present). Each scenario is adapted for each of the four types of cyberbullying behavior (Written—Verbal, Visual, Exclusion, Impersonation) resulting in a total number of 128 cyberbullying scenarios. We applied the same 128 scenarios to both cyberbullying and traditional bullying situations through 16 “leading cases,” specifying the cyber and the real context in different columns. In each column, we reported the sentence describing the presence or absence of the five criteria. For the purpose of the present study we used only data about cyberbullying. Examples and operationalization are presented in Tables 2–4. For each scenario of one type of cyberbullying (i.e., for 32 scenarios in total), participants were asked to rate on a 5-point Likert scale how severe it was (from *not severe* to *very severe*). In the Supplementary Material, the Scenarios Questionnaire is reported (i.e., leading cases and items for each cyberbullying behavior).

**Table 2:** Leading cases and sentences to connote the cyber context in the scenarios for each type of behavior.

	**In leading cases**	**CYBER**
WV	M. sent a nasty message	If the message was sent by internet or mobile phone
V	M. sent a compromising photo of C.	If the photo was sent by internet or mobile phone
E	M. excluded C. from their group	If the exclusion took place by internet or mobile phone
I	M. had got access to C.‘s private information	If M. had got access to the private information by internet or mobile phone

*WV, Written–Verbal behavior; V, Visual behavior; E, Exclusion behavior; I, Impersonation behavior*.

**Table 3:** Sentences defining the presence/absence of each criterion in the scenarios.

**Psychological Characteristic**		**Absent**	**Present**
Intentionality		It was a joke	M. wanted to hurt C. intentionally.
Imbalance of power		And C. didn't care.	C. was upset and didn't know how to defend him/herself.
Repetition		Once	Several times a month.
Public vs. Private	WV	To C.	About C. to other people.
	V	To C.	To other people
	E	Excluded C.	And other people had noticed it.
	I	To C.‘s *(private information)*	And he/she had shown it to other people.
Anonymity		To C., a boy/girl whom M. knows	C. didn't know who it was.

*Only for public vs. private criterion a different specification was needed for each type of behavior*.

**Table 4:** Examples of the phrasing and operationalization of scenarios for written-verbal behavior.

	**Leading case**	**Scenario for cyber context**
Scenario n°1—absence of criteria	“M. sent a nasty message to C., a boy/girl whom M. knows.”	If the message was sent once by internet or mobile phone, it was a joke and C. didn't care
Scenario n°21—presence of all criteria	“M. sent a nasty message about C. to other people. C. didn't know who it was.”	If the message was sent several times a month by internet or mobile phone, M. wanted to hurt C. intentionally and C. was upset and didn't know how to defend him/herself

Each set of 32 scenarios for each type of behavior was randomly assigned to different groups of participants in each school class, dividing the questionnaire administration into two parts and thus incorporating a break to prevent fatigue effects.

### Data analyses

We analyzed data using Exploratory Structural Equation Models (ESEM, Asparouhov and Muthén, 2009). The strict requirement of zero cross-loadings in Confirmatory Factor Analyses (CFA) did not fit our data well. Scenarios have been developed experimentally manipulating the presence/absence of criteria in all possible combinations and a better fitting approach seemed to be Exploratory Factor Analyses (EFA). However, an advantage of the ESEM is the possibility to test the measurement invariance for an EFA solution in relation to multiple groups (countries in our case), relaxing the CFA assumption that items have zero loadings on all factors other than the target factor, unrealistic for the Scenarios Questionnaire. In the EFA-SEM (ESEM) approach in addition to or instead of a CFA measurement model, an EFA measurement model with rotations can be used in a structural equation model. Restrictions for model identification are imposed by rotating the factor loading matrix and fixing the factor variances at one in one group.

Initially, we fitted the ESEM model (Geomin rotation) (Marsh et al., 2010) on 31 scenarios. As a result of the analyses made on the first version of the Scenarios Questionnaire (Menesini et al., 2012), we excluded scenario one (absence of all criteria) from further analyses also to avoid possible biases in the cross-national comparison of results^1^. The item is the first one administrated to the participants. It was used as an anchor to set the evaluation of severity of the other items (i.e., absence of every criteria). Considering the complexity of the Scenarios and the cognitive load requested of the participants to fill them out, we preferred to avoid possible bias due to the inclusion of the anchor item.

We compared models with a different number of factors (from 1 to 8) in order to detect the best trade-off between a parsimonious model and good fit indices.

Once the number of factors of the best solution had been identified, we examined the measurement invariance, that had to be demonstrated before concluding that the underlying factors are similar across countries. We followed the same steps for testing invariance in multiple group models (configural, metric, scalar variance-covariance, and latent mean invariance, Meredith, 1993; Muthén and Muthén, 1998-2012; Vandenberg and Lance, 2000; Marsh et al., 2010). We tested measurement invariance across countries comparing the models listed from the least (1st step) to the most restrictive (5th step). Specifically, the sequence of invariance testing begins with a model with all parameters freely estimated, and only the similarity of the overall pattern of parameters was evaluated (*A configural invariance–1st step*; Meredith, 1993). This provided both a test of the ability of the a priori model to fit the data in each group (country) without invariance constraints and a baseline for comparing the following constrained models. Weak measurement invariance (*B metric–2nd step*) was tested constraining as invariant the factor loadings over groups. Strong measurement invariance was satisfied if both the indicator means (i.e., the items' intercepts) and factor loadings were invariant over groups (*C scalar–3rd step*). In the next steps, we imposed equality of factors variance and the factor covariance (*D Factor variance–covariance invariance 4th step*) and lastly, we imposed the equality of the factor means (*E latent mean invariance 5th step*). Both for detecting the number of factors and for testing invariance, we selected the most appropriate model out of these sequences of models based firstly on an overall assessment of the RMSEA (Marsh et al., 2004) and CFI (Bollen, 1989). It has been suggested (Chen, 2007) that support for the more parsimonious model requires a change in CFI of < 0.01 and a change in RMSEA of < 0.015. We also considered the Bayesian Information Criterion (BIC) in testing for the evidence of invariance (Vrieze, 2012): lower BIC value indicates a better trade-off between fit and complexity.

Subsequently, we interpreted the invariant solution we found using a more stringent alpha level of 0.01 in evaluating the factor loadings as significant. In order to understand the meaning of these factors, we referred to the presence or absence of the criteria, and eventually patterns of criteria, in the scenarios that significantly load into the factors.

Finally, when there was no latent mean invariance, we analyzed country differences on the latent ESEM factorial scores using one-way ANOVAs. Effect size is evaluated by mean of eta squared (above 0.01 = small, above 0.06 = medium, above 0.13 = large; Cohen, 1988) and significant *post-hoc* analyses are reported using Tukey's test.

All the models were carried out using the Mplus computer program (Muthén and Muthén, 1998-2012; MLR estimator).

## Results

### Models fit

The ESEM models and their fit indices are presented in Table 5. The solution with five factors (Model 5 factors) can be considered the best trade-off between a parsimonious model and good fit indices. While having better fit indices, solutions with more factors are less parsimonious and lead to meaningless factors defined by only a few scenarios.

**Table 5:** Fit indices for ESEM models with factors from one to seven.

	**χ^2^**	**Df**	***P***	**CFI**	**RMSEA [90% C.I.] *probability* ≤0.05**	**BIC**	**N**
31 Scenarios ESEMs							1969
Model 1 Factor (a)	10352.635	434	0.000	0.602	0.108 [0.106–0.110] 0.000	164579.956	
Model 2 Factors (b)	5010.169	404	0.000	0.815	0.076 [0.074–0.078] 0.000	157182.543	
Model 3 Factors (c)	3532.629	375	0.000	0.873	0.065 [0.063–0.067] 0.000	155203.458	
Model 4 Factors (d)	2369.684	347	0.000	0.919	0.054 [0.052–0.056] 0.000	153784.455	
**Model 5 Factors (e)**	**1978.740**	**320**	**0.000**	**0.933**	**0.051 [0.049**–**0.053] 0.158**	**153295.210**	
Model 6 Factors (f)	1570.938	294	0.000	0.949	0.047 [0.045–0.049] 0.985	152927.559	
Model 7 Factors (g)	1189.578	269	0.000	0.963	0.042 [0.039–0.044] 1	152620.832	
Model 8 Factors (h)	952.555	245	0.000	0.972	0.038 [0.036–0.041] 1	152446.254	

*In bold the solution that can be considered the best trade-off between a parsimonious model and good fit indices*.

Subsequently, we tested for the invariance of this 5-factors model. In Table 6 the models' fit indices are reported for the comparison from the less restricted model (A–Configural Invariance: all parameters that are freely estimated) to the more constrained one (E–Latent Means Invariance: the factor loadings, the items intercepts, the factor covariance and the equality of the factor means are imposed over groups). Step by step introducing hierarchically the constrains, the overall fit indices, their change and BIC values led us to prefer the most restrictive model except for the final model E. In this case the increase in the BIC value suggested to reject this model. Consequently, the hypothesis of latent mean invariance has to be rejected. Overall we can say that the model was found to be invariant across countries, except for the latent factors' means.

**Table 6:** Testing invariance across countries for the ESEM 5-factor model: fit indices for the constrained models.

**5 FACTORS MODEL**		**Compared Model**		**χ^2^ (df)**	**CFI**	**ΔCFI**	**RMSEA [90% C.I.]**	**ΔRMSEA**	**BIC**
**INVARIANCE ACROSS COUNTRIES**									
A	Configural Inv.			3130.293 (1,280)	0.929		0.054 [0.052–0.057]		155934.864
B	Metric Inv.		A	3753.417 (1,670)	0.920	−0.009	0.050 [0.048–0.052]	−0.004	153818.803
C	Scalar Inv.		B	3995.194 (1,748)	0.914	−0.006	0.051 [0.049–0.053]	+0.001	153471.686
D	Variance-Covariance Inv.		C	4114.697 (1,793)	0.911	−0.003	0.051 [0.049–0.053]	0.000	153293.682
E	Latent Means Inv.		D	4313.846 (1,808)	0.904	−0.007	0.053 [0.051–0.055]	+0.002	153426.751

### Factors of perceived severity

The significant loadings and the pattern of presence/absence of criteria in each scenario are reported in Table 7 for Factors 1 and 2, Table 8 for Factor 3, Table 9 for Factor 4, and Table 10 for Factor 5. In the Supplementary Material, unstandardized factor loadings and standard errors for each of the five factors are reported.

**Table 7:** Factor 1 (Absence of Imbalance of Power) and Factor 2 (Presence of Imbalance of Power) – Unstandardized loadings and standard error and presence (P)/absence (A) of criteria.

**Scenario**	**Intention**	**Repetition**	**Imbalance of Power**	**Public/private**	**Anonymity**	**Factor 1**	**Factor 2**
16	P	P	**A**	PUB	A	**1.007 (0.031)**	−0.018 (0.024)
32	P	P	**A**	PRI	P	**0.993 (0.031)**	0.031 (0.026)
14	P	A	**A**	PUB	A	**0.981 (0.033)**	−0.028 (0.028)
15	A	P	**A**	PUB	A	**0.973 (0.046)**	−0.077 (0.041)
22	P	A	**A**	PUB	P	**0.969 (0.039)**	0.042 (0.042)
24	P	P	**A**	PUB	P	**0.969 (0.031)**	**0.078 (0.025)**
31	A	P	**A**	PRI	P	**0.956 (0.035)**	−**0.076 (0.026)**
23	A	P	**A**	PUB	P	**0.941 (0.042)**	−0.002 (0.025)
30	P	A	**A**	PRI	P	**0.926 (0.044)**	0.048 (0.048)
8	P	P	**A**	PRI	A	**0.847 (0.041)**	0.041 (0.026)
7	A	P	**A**	PRI	A	**0.830 (0.049)**	−0.039 (0.041)
25	A	A	**A**	PRI	P	**0.785 (0.034)**	−0.018 (0.023)
6	P	A	**A**	PRI	A	**0.784 (0.044)**	0.010 (0.025)
17	A	A	**A**	PUB	P	**0.720 (0.039)**	0.072 (0.034)
9	A	A	**A**	PUB	A	**0.716 (0.037)**	0.030 (0.020)
27	P	A	**P**	PRI	P	−0.016 (0.024)	**0.798 (0.042)**
19	P	A	**P**	PUB	P	−0.005 (0.021)	**0.786 (0.038)**
21	P	P	**P**	PUB	P	−0.009 (0.018)	**0.776 (0.034)**
20	A	P	**P**	PUB	P	0.011 (0.041)	**0.766 (0.043)**
18	A	A	**P**	PUB	P	0.021 (0.022)	**0.746 (0.037)**
3	P	A	**P**	PRI	A	−0.058 (0.032)	**0.738 (0.039)**
29	P	P	**P**	PRI	P	0.015 (0.016)	**0.730 (0.031)**
11	P	A	**P**	PUB	A	−0.008 (0.023)	**0.716 (0.040)**
4	A	P	**P**	PRI	A	−0.002 (0.014)	**0.701 (0.049)**
28	A	P	**P**	PRI	P	0.057 (0.036)	**0.699 (0.039)**
26	A	A	**P**	PRI	P	0.028 (0.020)	**0.699 (0.036)**
12	A	P	**P**	PUB	A	0.030 (0.037)	**0.690 (0.041)**
13	P	P	**P**	PUB	A	0.053 (0.021)	**0.688 (0.028)**
5	P	P	**P**	PRI	A	−0.015 (0.020)	**0.676 (0.035)**
10	A	A	**P**	PUB	A	0.0028 (0.021)	**0.652 (0.035)**
2	A	A	**P**	PRI	A	0.025 (0.027)	**0.575 (0.038)**

*In bold the significant loadings (p < 0.01). The boxes highlight the patterns of criteria that define the factors*.

**Table 8:** Factor 3 (Absence of Intentionality and Anonymity) − Unstandardized loadings and standard error and presence (P)/absence (A) of criteria.

**Scenario**	**Intention**	**Repetition**	**Imbalance of Power**	**Public/private**	**Anonymity**	**Factor 3**
4	**A**	P	P	PRI	**A**	**0.504 (0.155)**
2	**A**	A	P	PRI	**A**	**0.387 (0.054)**
7	**A**	P	A	PRI	**A**	**0.362 (0.108)**
12	**A**	P	P	PUB	**A**	**0.226 (0.045)**
10	**A**	A	P	PUB	**A**	**0.202 (0.079)**
19	**P**	A	P	PUB	**P**	−**0.165 (0.045)**
22	**P**	A	A	PUB	**P**	−**0.190 (0.072)**
27	**P**	A	P	PRI	**P**	−**0.246 (0.061)**
30	**P**	A	A	PRI	**P**	−**0.265 (0.071)**

*In bold the significant loadings (p < 0.01). The boxes highlight the patterns of criteria that define the factor*.

**Table 9:** Factor 4 (Absence of Intention and Repetition) – Unstandardized loadings and standard error and presence (P)/absence (A) of criteria.

**Scenario**	**Intention**	**Repetition**	**Imbalance of Power**	**Public/private**	**Anonymity**	**Factor 4**
17	**A**	**A**	A	PUB	P	**0.481 (0.047)**
9	**A**	**A**	A	PUB	A	**0.471 (0.054)**
18	**A**	**A**	P	PUB	P	**0.435 (0.052)**
10	**A**	**A**	P	PUB	A	**0.425 (0.046)**
26	**A**	**A**	P	PRI	P	**0.377 (0.058)**
25	**A**	**A**	A	PRI	P	**0.371 (0.059)**
2	**A**	**A**	P	PRI	A	**0.212 (0.064)**
11	**P**	**A**	P	PUB	A	**0.180 (0.070)**
16	**P**	**P**	A	PUB	A	−**0.218 (0.046)**
13	**P**	**P**	P	PUB	A	−**0.243 (0.055)**
32	**P**	**P**	A	PRI	P	−**0.249 (0.058)**
21	**P**	**P**	P	PUB	P	−**0.251 (0.080)**
24	**P**	**P**	A	PUB	P	−**0.268 (0.047)**
29	**P**	**P**	P	PRI	P	−**0.276 (0.075)**

*In bold the significant loadings (p < 0.01). The boxes highlight the patterns of criteria that define the factor*.

**Table 10:** Factor 5 (Anonymity and no Intention) – Unstandardized loadings and standard error and presence (P)/absence (A) of criteria.

**Scenario**	**Intention**	**Repetition**	**Imbalance of Power**	**Public/private**	**Anonymity**	**Factor 5**
23	**A**	P	A	PUB	**P**	**0.421 (0.054)**
31	**A**	P	A	PRI	**P**	**0.375 (0.043)**
25	**A**	A	A	PRI	**P**	**0.355 (0.090)**
26	**A**	A	P	PRI	**P**	**0.320 (0.087)**
28	**A**	P	P	PRI	**P**	**0.291 (0.058)**
20	**A**	P	P	PUB	**P**	**0.207 (0.078)**
13	**P**	P	P	PUB	**A**	−**0.165 (0.056)**
16	**P**	P	A	PUB	**A**	−**0.206 (0.064)**
11	**P**	A	P	PUB	**A**	−**0.267 (0.040)**
8	**P**	P	A	PRI	**A**	−**0.286 (0.090)**
14	**P**	A	A	PUB	**A**	−**0.320 (0.047)**
6	**P**	A	A	PRI	**A**	−**0.354 (0.077)**

*In bold the significant loadings (p < 0.01). The boxes highlight the patterns of criteria that define the factor*.

Factor 1 has excellent factor loadings and is clearly characterized by the scenarios where the criterion of imbalance of power is absent (“**absence of imbalance of power**”). Factor 2 has similar strong loadings and consists of all the scenarios where the criterion of imbalance of power is present (“**presence of imbalance of power**”). The only two exceptions are scenarios n° 24 and 31, which have very weak loading (0.078 and −0.076).

For Factor 3, the items' loadings are lower, ranging from −0.165 to 0.504; it is bidimensional and may be defined by the interplay between two criteria. On the positive extreme side, we find scenarios characterized by the *absence of intentionality and anonymity* and on the negative side by the *presence of both* characteristics. According to these features, it may be defined as “**absence of intention and anonymity**.”

The fourth factor's loadings range from 0.180 to 0.481. This factor is bidimensional and characterized by the *combination of intention and repetition*. Specifically, the scenarios in which intention and repetition are absent are on the positive pole of the dimension, while the presence of both intention and repetition is on the opposite pole. The only exception, out on 14 significant ones, is scenario number 11 for intention which have, however, quite weak loading (0.180)^2^. This factor can be defined as “**absence of intention and repetition**.”

Similarly to the third factor, the fifth factor is also characterized by anonymity and intentionality, but with a mixed presence/absence on the poles: on the positive side, scenarios are characterized by the *presence of anonymity contrasted with absence of intention* and, on the negative side, by the presence of intention contrasted by the absence of anonymity. This bidimensional factor (factors loadings ranging from −0.165 to 0.421) can be labeled “**anonymity and no intention**.”

### Country differences

**Figure 1:**
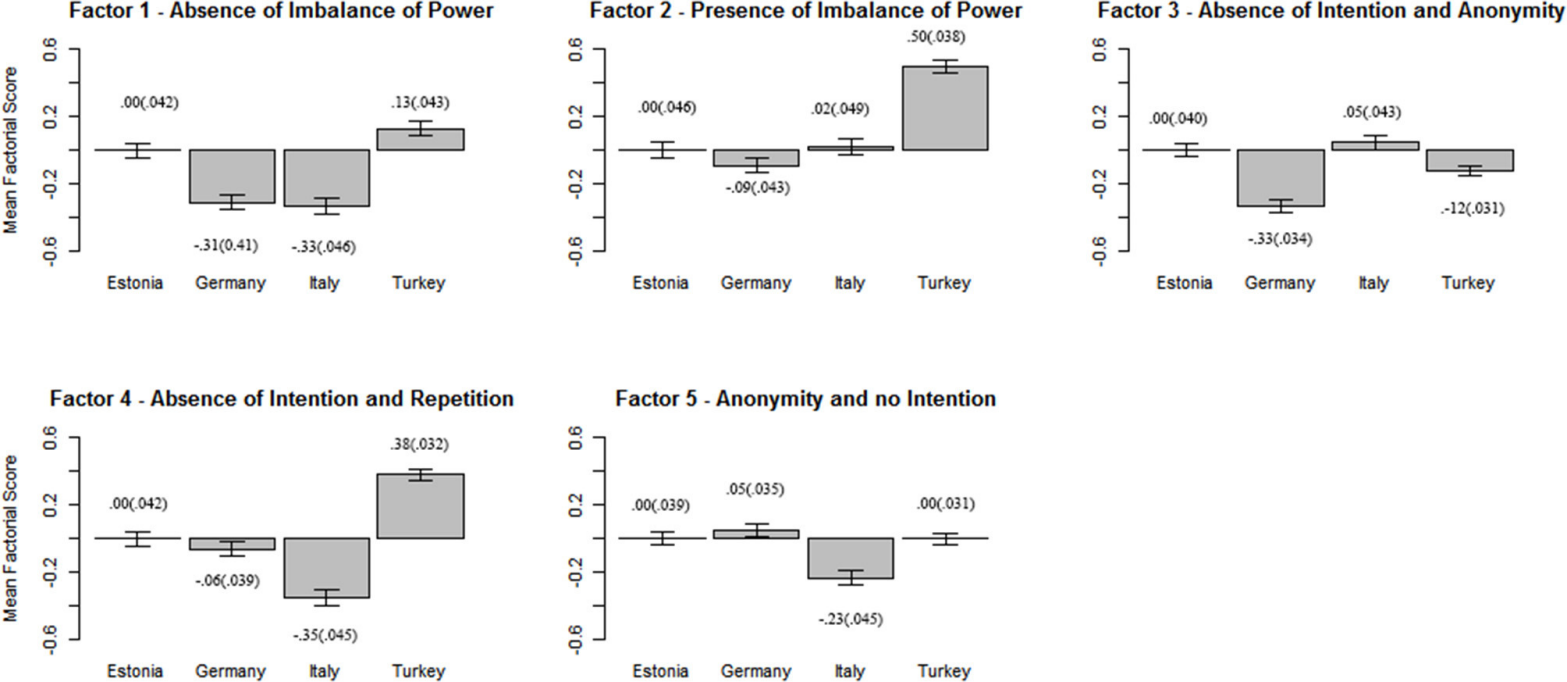
Factorial scores in each country. The Estonian mean score is displayed as the reference point. Error bars showed standard errors from the mean. Means and standard errors (in brackets) are displayed.

Given that the invariance of latent means was not found, we tested for the presence of cross-cultural differences. Looking at the results of the ANOVAs (Table 11), we found significant differences between countries with effect sizes ranging from small to medium. In Figure 1, the factorial scores and standard errors are reported for each country. Specifically looking at the *post-hoc* results, Factor 1 scores (absence of imbalance of power) were significantly lower in Italy and Germany compared to Estonia and Turkey; for Factor 2 (presence of imbalance of power) scores were higher in Turkey when compared to other countries (medium effect size). For Factor 3 (absence of intention and anonymity) we found the lowest score for Germany; Italy, had a higher score, followed by Germany and Turkey; finally, Estonia did not differ significantly from both Turkey and Italy. Particularly, when an attack is made by someone personally known without intention, German adolescents considered it as less severe. For factor 4 (absence of intention and repetition), Italy showed significantly lower factor scores when compared to the other countries. Germany and Estonia differed significantly from Turkey, that has the highest values on this factor (medium effect size). In summary, Italian adolescents stress the absence of intention and repetition as indicators of lower severity contrary to Turkish students. Finally, for Factor 5 (presence of anonymity and no intention) we found lower factor scores in Italy compared to the other countries. When the attack is not intentional and the actor is anonymous, Italian adolescents perceive these scenarios as less severe compared to adolescents of the other countries.

**Table 11:** Cross-cultural differences: ANOVAs results.

**Factor**	**ANOVAs**	***Post-Hoc* Results**
Factor 1 (Absence of imbalance of power)	*F*_(3, 1, 965)_ = 28.403, *p* < 0.01; η^2^ = 0.042	I G vs. E T
Factor 2 (Presence of imbalance of power)	*F*_(3, 1, 965)_ = 41.218, *p* < 0.01, η^2^ = 0.063	G E I vs. T
Factor 3 (Absence of intention and anonymity)	*F*_(3, 1, 965)_ = 13.749, *p* < 0.01, η^2^ = 0.031	G vs. T E vs. E I
Factor 4 (Absence of intention and repetition)	*F*_(3, 1, 965)_ = 0 43.868 *p* < 0.01, η^2^ = 0.083	I vs. G E vs. T
Factor 5 (Presence of anonymity and no intention)	*F*_(3, 1, 965)_ = 5.9147 *p* < 0.01, η^2^ = 0.014	I vs. T E G

*E, Estonia; G, Germany; I, Italy; T, Turkey. In the Post-Hoc Analyses column, the significant comparisons emerging from all the combinations are summarized*.

## Discussion

The present study was designed to determine the effects of different criteria on the perceived severity of cyberbullying incidents among adolescents. Results show that the criteria used by adolescents to rate scenarios of cyberbullying are the same across all four countries. Only the latent factors means of our model are not invariant, indicating that while the patterns are the same, the specific level of severity associated with each factor differs from country to country.

The most important criterion to define the severity of a cyberbullying scenario is imbalance of power. Specifically, the first two factors, namely the absence or presence of imbalance of power, are very strong and each show one clear dimension. They do not simply express a continuum in a bidimensional factor, but they show that if imbalance of power is present or not, this highly matters in terms of perceived severity. These findings confirm the strong role of imbalance of power for perception of severity in cyberbullying incidents, consistently with the results on studies about cyberbullying definition (Nocentini et al., 2010; Menesini et al., 2012; Dredge et al., 2014; Talwar et al., 2014). Indeed, recent studies found that participants identified power imbalance as the most relevant criterion when defining cyberbullying, followed by intentionality and anonymity.

The other criteria, such as intentionality, repetition, and anonymity are less relevant compared to imbalance of power and play a role only when combined with each other. Specifically, intentionality is present in three out of five factors, confirming the important role of this criterion in cyberbullying (Nocentini et al., 2010; Menesini et al., 2012), but it is always associated with another criterion. In Factors 3 and 4, intention is associated with anonymity and repetition, respectively, both in the same direction. As for Factor 5, intention is absent and anonymity is present. This confirms that anonymity might change the impact of a scenario in relation to the other criteria.

Contrary to previous studies, which considered repetition irrelevant for cyberbullying definition (Slonje and Smith, 2008; Dooley et al., 2009; Nocentini et al., 2010; Compton et al., 2014), the present study shows that repetition affects the perceived severity of the situation when it is combined with the intention to harm (Factor 4). This combined view of the criteria, is also stressed by authors focusing on the legal implications of cyberbullying (Langos, 2015).

In relation to publicity, we did not find any effect, contrary to the study by Sticca and Perren (2013). Sticca and Perren found that public cyberbullying was perceived as more severe than private but they did not evaluate the concurrent impact of imbalance of power, intentionality, repetition, and anonymity—or their possible combinations. They just considered private vs. public condition of cyberbullying. The difference between previous findings and ours can be explained by the fact that we used a more complex experimental design that took into consideration, simultaneously, the impact of all five criteria. Our results underlie that other dimensions (such as imbalance of power and intentionality) are more relevant when compared to the public/private dimension in determining the severity of the incidents while if considered alone and regardless of the other criteria, public vs. private nature of the attack can maintain its relevance.

The second aim was related to the investigation of cross-country differences. First of all, we have to highlight the invariance of the structure across the different samples meaning that the factors defining cyberbullying severity are perceived similarly across different countries. At the same time, the factor means are not invariant, meaning that some countries, compared to the others, attribute more importance to specific factors.

In terms of country-specific findings, Turkish participants perceive a scenario as more severe, compared to the other countries, when an imbalance of power is present (Factor 2). Given the recent attention to cyberbullying in Turkey, Turkish students are more sensitive overall to this criterion (Topçu et al., 2008). We can speculate that this difference might be related to cultural aspects such as power-related values (Schwartz et al., 2012), generally associated with social status and prestige, control or dominance over people and resources. At the same time, Turkish adolescents generally report higher scores in almost every factor compared to the other countries (see especially Factor 4). This may underline that Turkish students have a higher sensitivity to the attack *per se*. Consistently with these findings, in a qualitative study with students aged 12–18 years (Türkileri-İnselöz and Uçanok, 2013), almost half of the sample reported to perceive the negative impact of cyberbullying.

If the imbalance of power is not present, the cyberbullying scenarios are consistently less severe for Italian and German adolescents than for Turkish and Estonian adolescents (Factor 1). Thus, this criterion seems to be particularly important both for severity perception and for defining cyberbullying in these two countries (Menesini et al., 2012; Schultze-Krumbholz et al., 2014). When the scenarios do not present intentionality and anonymity (Factor 3), German adolescents perceive a lower level of severity. Italian adolescents, on the contrary, perceive less severity when there is anonymity and no intention (Factor 5). These results underline an important difference regarding anonymity. For Italians, an incident is more hurtful when it is led by a friend, even if it is not intentional. For German adolescents, the situation is scarier when the protagonist is someone that is not known personally. In a previous study based on focus groups involving German adolescents, Schultze-Krumbholz et al. (2014) found that anonymity and publicity are not necessary for the definition of cyberbullying, but they can impact the severity of the behavior. As reported by a German adolescent in a focus group “.it's actually disappointing when it's someone you trust and so on. However, on the other side it's bad if you don't know who it is because then, in principle, it could be anyone” (Nocentini et al., 2010). The present study partially confirms these findings indicating that anonymity matters more for German adolescents than for Italian adolescents.

At the same time when the scenarios do not include intentionality and repetition, Italian adolescents perceive a lower level of severity compared to the other countries (Factor 4). In a focus group study (Nocentini et al., 2010), Italian adolescents stressed the role of repetition, since this criterion can differentiate an intentional attack from a joke. So, although repetition is not a criterion necessary for the definition of cyberbullying, in the present study about severity it plays a role together with intentionality for Italian participants.

To summarize, Turkish adolescents have a stronger focus on imbalance of power but they also report a higher level of severity in general compared to adolescents of the other countries. For Italian participants, an anonymous non-intentional attack is less threatening than in other countries. On the contrary, for German adolescents, not knowing the perpetrator may cause insecurity and fear. Nevertheless, the role of anonymity in cyberbullying is controversial. For some authors, this criterion simply differentiates the face-to-face context from the virtual context (Dooley et al., 2009; Sticca and Perren, 2013; Patchin and Hinduja, 2015); for others, the supposed features of anonymity may encourage young people to cyberbully (Hoff and Mitchell, 2009).

Finally, Estonian adolescents have similar scores to adolescents from Turkey with regard to the absence of imbalance of power, and generally show intermediate factorial scores when compared to the adolescents of the other countries.

### Limitations and strengths

One limitation of this study is that the data refer to different types of cyberbullying behavior, but these types were not included in the analysis. Specifically, we did not take into direct consideration the analyses of the different types of cyberbullying because of the complexity of the research design and the Scenarios. At the moment, the statistical models cannot allow us to consider both aspects at the same time in a unique model. More attention to this aspect of different perceived severity related to different types of attacks looks very promising both from the psychological and legal points of view and needs further investigation (Nocentini et al., 2010; Menesini et al., 2012; Langos, 2015).

Another issue is related to the presence/absence of “anonymity.” In the definition of anonymity we stated that the bully attacks the victim who does not know his or her identities. For the opposite condition of absence of anonymity, we stated that the bully knows the victims, but we did not directly clarify if the victim recognized the perpetrator or not. This operationalization was used as a better tool to improve the understanding of the sentences that diversely would have been too long and complex. This point does not affect the role of anonymity which is clearly defined but it might have an impact on the absence of anonymity and maybe on Factor 3. Further studies can clarify whether familiarity and knowledge between bullies and victims stated in our scenarios is enough to define the absence of anonymity or if it is necessary to clearly express the point of view of the victim.

Furthermore, given our general aim on perceived severity, we focused on the whole population of adolescents, without considering adolescents' roles in the cyberbullying dynamics. There might be significant differences in the perceived severity among perpetrators, victims, bully/victims, and non-involved individuals, and we hope that future studies can explore this dimension further. Bullying is conceptualized as a group phenomenon often involving a large group of peers. In addition to the main roles of bullies and victims, other children may play a role in the bullying dynamics, acting as a reinforcer or assistant of the bully, as outsider or passive bystanders and more rarely as a defender of the victim (Salmivalli et al., 1996). In the last 10 years, some scholars have explored the adaptation of the participant role model to the cyber space (Bastiaensens et al., 2014). Cyberbullying incidents often occur in the presence of other bystanders, those who witness cyberbullying incidents. For this group of persons a variety of reactions can be found (e.g., defending the victim, telling the bully to stop, ignoring what was going on, spreading and disseminating the message, up to the point of directly joining in on the harassment). The construct of perceived severity can be highly relevant to understanding the behaviors of bystanders in the peer group (Salmivalli, 2010) and to determine whether and to what extent the perception of the situation may enhance the probability of defending the victim even in the cyber context (Bastiaensens et al., 2014). In fact, understanding the relation between the perception of severity and the suffering of the victims (e.g., empathy, Machackova and Pfetsch, 2016) may be relevant to promote defending behaviors among bystanders in the cyber context.

Finally, we focused on country differences rather than cultural differences, since we did not take into account different cultural elements that could have influenced adolescents' perception. The present study is the first step in understanding cultural-specific aspects related to adolescents' perception of cyberbullying severity. Future studies could include variables such as socio-moral development, human values (Schwartz et al., 2012), socio-economic aspects etc. to better explain the differences between countries. Such investigations will allow us to better understand the interplay between individual and cultural aspects in the perceived severity of cyberbullying.

Despite these limitations, the results of this research support the idea that the perception of severity is related to defining criteria and characteristics of cyberbullying. Considering together perceived severity and cyberbullying definition, we should recognize the role of imbalance of power and to a lower extent of intentionality and anonymity. These findings support recent contributions in the area of evolutionary psychology, which defines bullying as aggressive goal directed behavior causing harm to another individual within the context of a power imbalance (Volk et al., 2017). From the perpetrator's point of view, one motivational factor is to reach social and material goals. From the victims' point of view, the dynamics are more complex. If the bully attacks and the victim is upset and does not know how to defend him/herself, then this interactional dynamic, together with the initial differential, enhances the imbalance within the dyad and, in turn, the perception of seriousness of the cyberbullying attack. Our definition of imbalance of power focuses on the victim's reaction and on his/her status in the relationship. It also introduce a dynamic description of the process starting from the initial level of goal achievement and dominance attributed to bullies. Additionally, it underscores the role of the dynamic process between the actor, the victim and the bystanders in the process (Menesini et al., 2012).

In light of these findings, intervention and prevention strategies might address (potential) victims' assertiveness and social resources, reducing their feelings of helplessness and inferiority. On the other side, bullies should be supported in finding positive ways to express their motivation for dominance and bystanders should be encouraged to offer their support to victims in order to reduce the perceived imbalance of power in the relationship.

## Ethics statements

The research was carried out in accordance with the Ethic Research Recommendations of the Italian Association of Psychology. It was not approved by a Ethical Research Committee because at that time (2012–2013) the University of Florence did not have an Ethical Committee, neither the approval was specifically requested by the Italian Association of Psychology. In each country, the research was carried out with the maximum respect of the participants and in accordance with the Declaration of Helsinki. Students or parents' written consent was obtained following the country law. Specifically, in Italy and Estonia school', parents', and students' active consent were obtained prior to conduct the questionnaires administration. In Germany active parental consent was acquired for students under 14 years of age (in accordance with the Senate regulations for empirical studies in the school setting) and students' active consent was collected in the first step of the questionnaire. In Turkey the research team requested a specific permission from the Ministry of Education and from school administrations. Participation was entirely voluntary, confidential and anonymous. Participants were all informed that they could withdraw from the study at any time. Specific ethical approval was not required for this type of study in accordance with the legislation and guidelines in Germany, Italy, Estonia, and Turkey. The research was carried out in accordance with the Ethic Research Recommendations of the Italian Association of Psychology.

## Author contributions

All authors listed have made substantial, direct, and intellectual contribution to the work, and approved it for publication.

### Conflict of interest statement

The authors declare that the research was conducted in the absence of any commercial or financial relationships that could be construed as a potential conflict of interest.

## Footnotes

^1^Scenarios number one showed consistently lower mean as compared to the others. Despite this general trend, we found differences between the countries: Italy (*M* = 2.12; *SD* = 1.04) and Germany (*M* = 2.14, *SD* = 1.13) reported lower scores compared to Turkey (*M* = 2.67; *SD* = 1.44) and Estonia (*M* = 2.79; *SD* = 1.215)—one-way ANOVA [*F*_(3, 1, 965)_ = 35.426, *p* < 0.01; η^2^ = 0.05].

^2^Given the strong relations between items, factors and the combination of presence/absence of criteria in each scenario, we can argue that probably there are other, not fundamental, combinations of criteria that played a secondary role in determining severity.
