# Supplementary material for: Perceived Severity of Cyberbullying: Differences and Similarities across Four Countries
Source: Front Psychol. 2017 Sep 20;8:1524. doi: 10.3389/fpsyg.2017.01524 (PMC5611493; doi:10.3389/fpsyg.2017.01524)
Supplement: Supplementary file 1 [file DataSheet1.docx]

**Supplementary Material – The Scenarios Questionnaire: Leading Cases and items for each cyberbullying behaviour (Exclusion, Impersonation, Visual and Written Verbal)**

| **EXCLUSION** | |
| --- | --- |
| **CASE 1E:**  **M. *excluded* C., a boy/girl whom M. Knows, *from their group*.** | |
|  | If the exclusion took place once **by internet or mobile phone**, it was a joke and C. didn’t care. |
|  | If the exclusion took place once **by internet or mobile phone**, it was a joke and C. was upset and didn’t know how to defend him/herself. |
|  | If the exclusion took place once **by internet or mobile phone**, M. wanted to hurt C. intentionally and C. was upset and didn’t know how to defend him/herself. |
|  | If the exclusion took place several times a month **by internet or mobile phone**, it was a joke and C. was upset and didn’t know how to defend him/herself. |
|  | If the exclusion took place several times a month **by internet or mobile phone**, M. wanted to hurt C. intentionally and C. was upset and didn’t know how to defend him/herself. |
|  | If the exclusion took place once **by internet or mobile phone**, M. wanted to hurt C. intentionally and C. didn’t care. |
|  | If the exclusion took place several times a month **by internet or mobile phone,** it was a joke and C. didn’t care. |
|  | If the exclusion took place several times a month **by internet or mobile phone**, M. wanted to hurt C. intentionally and C. didn’t care. |
| **CASE 2E:**  **M. *excluded* *C.,* a boy/girl whom M. knows, *from their* *group* and other people had noticed it.** | |
|  | If the exclusion took place once **by internet or mobile phone**, it was a joke and C. didn’t care. |
|  | If the exclusion took place once **by internet or mobile phone**, it was a joke and C. was upset and didn’t know how to defend him/herself. |
|  | If the exclusion took place once **by internet or mobile phone**, M. wanted to hurt C. intentionally and C. was upset and didn’t know how to defend him/herself. |
|  | If the exclusion took place several times a month **by internet or mobile phone**, it was a joke and C. was upset and didn’t know how to defend him/herself. |
|  | If the exclusion took place several times a month **by internet or mobile phone**, M. wanted to hurt C. intentionally and C. was upset and didn’t know how to defend him/herself. |
|  | If the exclusion took place once **by internet or mobile phone**, M. wanted to hurt C. intentionally and C. didn’t care. |
|  | If the exclusion took place several times a month **by internet or mobile phone,** it was a joke and C. didn’t care. |
|  | If the exclusion took place several times a month **by internet or mobile phone**, M. wanted to hurt C. intentionally and C. didn’t care. |
| **CASE 3E:**  **M. *excluded* C. *from their* *group* and other people had noticed it. C. didn’t know who it was.** | |
|  | If the exclusion took place once **by internet or mobile phone**, it was a joke and C. didn’t care. |
|  | If the exclusion took place once **by internet or mobile phone**, it was a joke and C. was upset and didn’t know how to defend him/herself. |
|  | If the exclusion took place once **by internet or mobile phone**, M. wanted to hurt C. intentionally and C. was upset and didn’t know how to defend him/herself. |
|  | If the exclusion took place several times a month **by internet or mobile phone**, it was a joke and C. was upset and didn’t know how to defend him/herself. |
|  | If the exclusion took place several times a month **by internet or mobile phone**, M. wanted to hurt C. intentionally and C. was upset and didn’t know how to defend him/herself. |
|  | If the exclusion took place once **by internet or mobile phone**, M. wanted to hurt C. intentionally and C. didn’t care. |
|  | If the exclusion took place several times a month **by internet or mobile phone,** it was a joke and C. didn’t care. |
|  | If the exclusion took place several times a month **by internet or mobile phone**, M. wanted to hurt C. intentionally and C. didn’t care. |
| **CASE 4E:**  **M. *excluded* C. *from their* *group*. C. didn´t know who it was.** | |
|  | If the exclusion took place once **by internet or mobile phone**, it was a joke and C. didn’t care. |
|  | If the exclusion took place once **by internet or mobile phone**, it was a joke and C. was upset and didn’t know how to defend him/herself. |
|  | If the exclusion took place once **by internet or mobile phone**, M. wanted to hurt C. intentionally and C. was upset and didn’t know how to defend him/herself. |
|  | If the exclusion took place several times a month **by internet or mobile phone**, it was a joke and C. was upset and didn’t know how to defend him/herself. |
|  | If the exclusion took place several times a month **by internet or mobile phone**, M. wanted to hurt C. intentionally and C. was upset and didn’t know how to defend him/herself. |
|  | If the exclusion took place once **by internet or mobile phone**, M. wanted to hurt C. intentionally and C. didn’t care. |
|  | If the exclusion took place several times a month **by internet or mobile phone,** it was a joke and C. didn’t care. |
|  | If the exclusion took place several times a month **by internet or mobile phone**, M. wanted to hurt C. intentionally and C. didn’t care. |
| **IMPERSONATION** | |
| **Case 1I:**  **M. *had got access* to *C.`s private information*, a boy/girl whom M. knows.** | |
|  | If M. had got access to the private information **by internet or mobile phone,** it happened once, it was a joke and C. didn’t care. |
|  | If M. had got access to the private information **by internet or mobile phone**, it happened once, it was a joke and C. was upset and didn’t know how to defend him/herself. |
|  | If M. had got access to the private information **by internet or mobile phone**, it happened once, M. wanted to hurt C. intentionally and C. was upset and didn’t know how to defend him/herself. |
|  | If M. had got access to the private information **by internet or mobile phone**, it happened several times a month**,** it was a joke and C. was upset and didn’t know how to defend him/herself. |
|  | If M. had got access to the private information **by internet or mobile phone**, it happened several times a month, M. wanted to hurt C. intentionally and C. was upset and didn’t know how to defend him/herself. |
|  | If M. had got access to the private information **by internet or mobile phon**e, it happened once, M. wanted to hurt C. intentionally and C. didn’t care. |
|  | If M. had got access to the private information **by internet or mobile phone**, it happened several times a month, it was a joke and C. didn’t care. |
|  | If M. had got access to the private information **by internet or mobile phone**, it happened several times a month, M. wanted to hurt C. intentionally and C. didn’t care. |
| **Case 2I:**  **M. *had got access to C`s private information*, a boy/girl whom M. knows, and he/she had shown it to other people.** | |
|  | If M. had got access to the private information **by internet or mobile phone,** it happened once, it was a joke and C. didn’t care. |
|  | If M. had got access to the private information **by internet or mobile phone**, it happened once, it was a joke and C. was upset and didn’t know how to defend him/herself. |
|  | If M. had got access to the private information **by internet or mobile phone**, it happened once, M. wanted to hurt C. intentionally and C. was upset and didn’t know how to defend him/herself. |
|  | If M. had got access to the private information **by internet or mobile phone**, it happened several times a month**,** it was a joke and C. was upset and didn’t know how to defend him/herself. |
|  | If M. had got access to the private information **by internet or mobile phone**, it happened several times a month, M. wanted to hurt C. intentionally and C. was upset and didn’t know how to defend him/herself. |
|  | If M. had got access to the private information **by internet or mobile phone**, it happened once, M. wanted to hurt C. intentionally and C. didn’t care. |
|  | If M. had got access to the private information **by internet or mobile phone**, it happened several times a month, it was a joke and C. didn’t care. |
|  | If M. had got access to the private information by **internet or mobile phone**, it happened several times a month, M. wanted to hurt C. intentionally and C. didn’t care. |
| **Case 3I:**  **M. *had got access to C.`s private information* and he/she had shown it to other people. C. didn’t know who it was.** | |
|  | If M. had got access to the private information **by internet or mobile phone,** it happened once, it was a joke and C. didn’t care. |
|  | If M. had got access to the private information **by internet or mobile phone**, it happened once, it was a joke and C. was upset and didn’t know how to defend him/herself. |
|  | If M. had got access to the private information **by internet or mobile phone**, it happened once, M. wanted to hurt C. intentionally and C. was upset and didn’t know how to defend him/herself. |
|  | If M. had got access to the private information **by internet or mobile phone**, it happened several times a month**,** it was a joke and C. was upset and didn’t know how to defend him/herself. |
|  | If M. had got access to the private information **by internet or mobile phone**, it happened several times a month, M. wanted to hurt C. intentionally and C. was upset and didn’t know how to defend him/herself. |
|  | If M. had got access to the private information **by internet or mobile phon**e, it happened once, M. wanted to hurt C. intentionally and C. didn’t care. |
|  | If M. had got access to the private information **by internet or mobile phone**, it happened several times a month, it was a joke and C. didn’t care. |
|  | If M. had got access to the private information **by internet or mobile phone**, it happened several times a month, M. wanted to hurt C. intentionally and C. didn’t care. |
| **Case 4I:**  **M. *had got access to C.`s private information*. C. didn’t know who it was.** | |
|  | If M. had got access to the private information **by internet or mobile phone,** it happened once, it was a joke and C. didn’t care. |
|  | If M. had got access to the private information **by internet or mobile phone**, it happened once, it was a joke and C. was upset and didn’t know how to defend him/herself. |
|  | If M. had got access to the private information **by internet or mobile phone**, it happened once, M. wanted to hurt C. intentionally and C. was upset and didn’t know how to defend him/herself. |
|  | If M. had got access to the private information **by internet or mobile phone**, it happened several times a month**,** it was a joke and C. was upset and didn’t know how to defend him/herself. |
|  | If M. had got access to the private information **by internet or mobile phone**, it happened several times a month, M. wanted to hurt C. intentionally and C. was upset and didn’t know how to defend him/herself. |
|  | If M. had got access to the private information **by internet or mobile phone**, it happened once, M. wanted to hurt C. intentionally and C. didn’t care. |
|  | If M. had got access to the private information **by internet or mobile phone**, it happened several times a month, it was a joke and C. didn’t care. |
|  | If M. had got access to the private information by **internet or mobile phone**, it happened several times a month, M. wanted to hurt C. intentionally and C. didn’t care. |
| **VISUAL** | |
| **CASE 1V:**  **M. *sent* a *compromising photo* of C. to C., a boy/girl whom M. knows.** | |
|  | If the photo was sent once **by internet or mobile phon**e, it was a joke and C. didn’t care. |
|  | If the photo was sent once **by internet or mobile phone**, it was a joke and C. was upset and didn’t know how to defend him/herself. |
|  | If the photo was sent once **by internet or mobile phone**, M. wanted to hurt C. intentionally and C. was upset and didn’t know how to defend him/herself. |
|  | If the photo was sent several times a month **by internet or mobile phone**, it was a joke and C. was upset and didn’t know how to defend him/herself. |
|  | If the photo was sent several times a month **by internet or mobile phone**, M. wanted to hurt C. intentionally and C. was upset and didn’t know how to defend him/herself. |
|  | If the photo was sent once **by internet or mobile phone**, M. wanted to hurt C. intentionally and C . didn’t |
|  | If the photo was sent several times a month **by internet or mobile phone**, it was a joke and C. didn’t care |
|  | If the photo was sent several times a month **by internet or mobile phone**, M. wanted to hurt C. intentionally and C. didn’t care. |
| **CASE 2V:**  **M. *sent* a *compromising photo of C.,* a boy/girl whom M. knows, to other people*.*** | |
|  | If the photo was sent once **by internet or mobile phon**e, it was a joke and C. didn’t care. |
|  | If the photo was sent once **by internet or mobile phone**, it was a joke and C. was upset and didn’t know how to defend him/herself. |
|  | If the photo was sent once **by internet or mobile phone**, M. wanted to hurt C. intentionally and C. was upset and didn’t know how to defend him/herself. |
|  | If the photo was sent several times a month **by internet or mobile phone**, it was a joke and C. was upset and didn’t know how to defend him/herself. |
|  | If the photo was sent several times a month **by internet or mobile phone**, M. wanted to hurt C. intentionally and C. was upset and didn’t know how to defend him/herself. |
|  | If the photo was sent once **by internet or mobile phone**, M. wanted to hurt C. intentionally and C. didn’t care. |
|  | If the photo was sent several times a month **by internet or mobile phone**, it was a joke and C. didn’t care |
|  | If the photo was sent several times a month **by internet or mobile phone**, M. wanted to hurt C. intentionally and C. didn’t care. |
| **CASE 3V:**  **M. *sent* a *compromising photo of C*. to other people. C. didn’t know who it was.** | |
|  | If the photo was sent once **by internet or mobile phon**e, it was a joke and C. didn’t care. |
|  | 1. If the photo was sent once **by internet or mobile phone**, it was a joke and C. was upset and didn’t know how to defend him/herself. |
|  | 1. If the photo was sent once **by internet or mobile phone**, M. wanted to hurt C. intentionally and C. was upset and didn’t know how to defend him/herself. |
|  | 1. If the photo was sent several times a month **by internet or mobile phone**, it was a joke and C. was upset and didn’t know how to defend him/herself. |
|  | If the photo was sent several times a month **by internet or mobile phone**, M. wanted to hurt C. intentionally and C. was upset and didn’t know how to defend him/herself. |
|  | If the photo was sent once **by internet or mobile phone**, M. wanted to hurt C. intentionally and C . didn’t care. |
|  | If the photo was sent several times a month **by internet or mobile phone**, it was a joke and C. didn’t care |
|  | If the photo was sent several times a month **by internet or mobile phone**, M. wanted to hurt C. intentionally and C. didn’t care. |
| **CASE 4V:**  **M. *sent* a *compromising photo* of C. to C. C. didn’t know who it was.** | |
|  | If the photo was sent once **by internet or mobile phon**e, it was a joke and C. didn’t care. |
|  | If the photo was sent once **by internet or mobile phone**, it was a joke and C. was upset and didn’t know how to defend him/herself. |
|  | If the photo was sent once **by internet or mobile phone**, M. wanted to hurt C. intentionally and C. was upset and didn’t know how to defend him/herself. |
|  | If the photo was sent several times a month **by internet or mobile phone**, it was a joke and C. was upset and didn’t know how to defend him/herself. |
|  | If the photo was sent several times a month **by internet or mobile phone**, M. wanted to hurt C. intentionally and C. was upset and didn’t know how to defend him/herself. |
|  | If the photo was sent once **by internet or mobile phone**, M. wanted to hurt C. intentionally and C. didn’t care. |
|  | If the photo was sent several times a month **by internet or mobile phone**, it was a joke and C. didn’t care |
|  | If the photo was sent several times a month **by internet or mobile phone**, M. wanted to hurt C. intentionally and C. didn’t care. |
| **WRITTEN VERBAL** | |
| CASE 1WV:  M. *sent* a *nasty message* to C., a boy/girl whom M. knows. | |
|  | If the message was sent once **by internet or mobile phone**, it was a joke and C. didn’t care. |
|  | If the message was sent once **by internet or mobile phone**, it was a joke and C. was upset and didn’t know how to defend him/herself. |
|  | If the message was sent once **by internet or mobile phone**, M. wanted to hurt C. intentionally and C. was upset and didn’t know how to defend him/herself. |
|  | If the message was sent several times a month **by internet or mobile phone**, it was a joke and C. was upset and didn’t know how to defend him/herself. |
|  | If the message was sent several times a month **by internet or mobile phone**, M. wanted to hurt C. intentionally and C. was upset and didn’t know how to defend him/herself. |
|  | If the message was sent once **by internet or mobile phone**, M. wanted to hurt C. intentionally and C. didn’t care. |
|  | If the message was sent several times a month **by internet or mobile phone**, it was a joke and C. didn’t care. |
|  | If the message was sent several times a month **by internet or mobile phone**, M. wanted to hurt C. intentionally and C. didn’t care. |
| **Case 2WV:**  **M. *sent* a *nasty message about C.*, a boy/girl whom M. knows, to other people.** | |
|  | If the message was sent once **by internet or mobile phone**, |
|  | it was a joke and C. didn’t care. |
|  | If the message was sent once **by internet or mobile phone**, it was a joke and C. was upset and didn’t know how to defend him/herself. |
|  | If the message was sent once **by internet or mobile phone**, M. wanted to hurt C. intentionally and C. was upset and didn’t know how to defend him/herself. |
|  | If the message was sent several times a month |
|  | **by internet or mobile phone**, it was a joke and C. was upset and didn’t know how to defend him/herself. |
|  | If the message was sent several times a month |
|  | **by internet or mobile phone**, M. wanted to hurt C. intentionally and C. was upset and didn’t know how to defend him/herself. |
| CASE 3WV:  M. *sent* a *nasty message* about C. to other people. C. didn´t know who it was. | |
|  | If the message was sent once **by internet or mobile phone**, it was a joke and C. didn’t care. |
|  | If the message was sent once **by internet or mobile phone**, it was a joke and C. was upset and didn’t know how to defend him/herself. |
|  | If the message was sent once **by internet or mobile phone**, M. wanted to hurt C. intentionally and C. was upset and didn’t know how to defend him/herself. |
|  | If the message was sent several times a month **by internet or mobile phone**, it was a joke and C. was upset and didn’t know how to defend him/herself. |
|  | If the message was sent several times a month **by internet or mobile phone**, M. wanted to hurt C. intentionally and C. was upset and didn’t know how to defend him/herself. |
|  | If the message was sent once **by internet or mobile phone**, M. wanted to hurt C. intentionally and C. didn’t care. |
|  | If the message was sent several times a month **by internet or mobile phone**, it was a joke and C. didn’t care. |
|  | If the message was sent several times a month **by internet or mobile phone**, M. wanted to hurt C. intentionally and C. didn’t care. |
| CASE 4WV:  M. *sent* a *nasty message* to C. C. didn´t know who it was. | |
|  | If the message was sent once **by internet or mobile phone**, it was a joke and C. didn’t care. |
|  | If the message was sent once **by internet or mobile phone**, it was a joke and C. was upset and didn’t know how to defend him/herself. |
|  | If the message was sent once **by internet or mobile phone**, M. wanted to hurt C. intentionally and C. was upset and didn’t know how to defend him/herself. |
|  | If the message was sent several times a month **by internet or mobile phone**, it was a joke and C. was upset and didn’t know how to defend him/herself. |
|  | If the message was sent several times a month **by internet or mobile phone**, M. wanted to hurt C. intentionally and C. was upset and didn’t know how to defend him/herself. |
|  | If the message was sent once **by internet or mobile phone**, M. wanted to hurt C. intentionally and C. didn’t care. |
|  | If the message was sent several times a month **by internet or mobile phone**, it was a joke and C. didn’t care. |
|  | If the message was sent several times a month **by internet or mobile phone**, M. wanted to hurt C. intentionally and C. didn’t care. |

**Supplementary Material – Final 5-factor model (Variance-Covariance Invariance) unstandardized factor loadings and standard errors**

| Item | Factor 1 | Factor 2 | Factor 3 | Factor 4 | Factor 5 |
| --- | --- | --- | --- | --- | --- |
| S2 | .025 (.027) | **.575 (.038)** | **.387 (.054)** | **.212 (.064)** | -.006 (.043) |
| S3 | -.058 (.032) | **.738 (.039)** | .268 (.108) | -.009 (.049) | -.083 (.083) |
| S4 | -.002 (.014) | **.701 (.049)** | **.504 (.155)** | -.187 (.072) | .154 (.107) |
| S5 | -.015 (.020) | **.676 (.035)** | .209 (.136) | -.313 (.048) | -.085 (.095) |
| S6 | **.784 (.044)** | .010 (.025) | .126 (.093) | .029 (.050) | **-.354 (.077)** |
| S7 | **.830 (.049)** | -.039 (.041) | **.362 (.108)** | .052 (.092) | -.068 (.117) |
| S8 | **.847 (.041)** | .041 (.026) | .157 (.080) | -.133 (.062) | **-.286 (.090)** |
| S9 | **.716 (.037)** | .030 (.020) | .185 (.137) | **.471 (.054)** | -.100 (.099) |
| S10 | .028 (.021) | **.652 (.035)** | **.202 (.079)** | **.425 (.046)** | -.052 (.058) |
| S11 | -.008 (.023) | **.716 (.040)** | -.031 (.048) | **.180 (.070)** | **-.267 (.040)** |
| S12 | .030 (.037) | **.690 (.041)** | **.226 (.045)** | .041 (.050) | .051 (.071) |
| S13 | .053 (.021) | **.688 (.028)** | .000 (.064) | **-.243 (.055)** | **-.165 (.056)** |
| S14 | **.981 (.033)** | -.028 (.028) | -.029 (.055) | .037 (.042) | **-.320 (.047)** |
| S15 | **.973 (.046)** | -.077 (.041) | .182 (.083) | .041 (.062) | .038 (.103) |
| S16 | **1.007 (.031)** | -.018 (.024) | .017 (.031) | **-.218 (.046)** | **-.206 (.064)** |
| S17 | **.720 (.039)** | .072 (.034) | -.036 (.066) | **.481 (.047)** | .175 (.119) |
| S18 | .021 (.022) | **.746 (.037)** | -.034 (.049) | **.435 (.052)** | .141 (.110) |
| S19 | -.005 (.021) | **.786 (.038)** | **-.165 (.045)** | .102 (.073) | -.105 (.056) |
| S20 | .011 (.041) | **.766 (.043)** | .106 (.064) | -.008 (.036) | **.207 (.078)** |
| S21 | -.009 (.018) | **.776 (.034)** | -.196 (.096) | **-.251 (.080)** | -.058 (.043) |
| S22 | **.969 (.039)** | .042 (.042) | **-.190 (.072)** | -.005 (.066) | .000 (.053) |
| S23 | **.941 (.042)** | -.002 (.025) | .096 (.063) | .002 (.041) | **.421 (.054)** |
| S24 | **.969 (.031)** | **.078 (.025)** | -.106 (.095) | **-.268 (.047)** | .087 (.049) |
| S25 | **.785 (.034)** | -.018 (.023) | -.023 (.027) | **.371 (.059)** | **.355 (.090)** |
| S26 | .028 (.020) | **.699 (.036)** | -.026 (.029) | **.377 (.058)** | **.320 (.087)** |
| S27 | -.016 (.024) | **.798 (.042)** | **-.246 (.061)** | .081 (.083) | .020 (.050) |
| S28 | .057 (.036) | **.699 (.039)** | .027 (.053) | -.027 (.037) | **.291 (.058)** |
| S29 | .015 (.016) | **.730 (.031)** | -.222 (.096) | **-.276 (.075)** | .028 (.028) |
| S30 | **.926 (.044)** | .048 (.048) | **-.265 (.071)** | .011 (.075) | -.021 (.052) |
| S31 | **.956 (.035)** | **-.076 (.026)** | -.001 (.042) | .029 (.043) | **.375 (.043)** |
| S32 | **.993 (.031)** | .031 (.026) | -.175 (.098) | **-.249 (.058)** | .059 (.044) |

*Note*: In bold the significant loadings (p<.01); standard errors in brackets
